# Extracorporeal life support with left ventricular decompression—improved survival in severe cardiogenic shock: results from a retrospective study

**DOI:** 10.7717/peerj.3813

**Published:** 2017-09-29

**Authors:** Bastian Schmack, Philipp Seppelt, Alexander Weymann, Christina Alt, Mina Farag, Rawa Arif, Andreas O. Doesch, Philip W. Raake, Klaus Kallenbach, Ashham Mansur, Aron-Frederik Popov, Matthias Karck, Arjang Ruhparwar

**Affiliations:** 1 Department of Cardiac Surgery, University Hospital Heidelberg, Heidelberg, Germany; 2 Medical Clinic III, Department of Cardiology, University Hospital Frankfurt, Frankfurt, Germany; 3 Department of Cardiac Surgery, University Hospital Oldenburg, Oldenburg, Germany; 4 Department of Cardiology, University Hospital Heidelberg, Heidelberg, Germany; 5 Department of Cardiac Surgery, INCCI National Heart Institute, Luxembourg, Luxembourg; 6 Department of Anesthesiology, Emergency and Intensive Care Medicine, University Medical Center, University of Goettingen, Goettingen, Germany; 7 Division of Thoracic and Cardiovascular Surgery, University Hospital Frankfurt, Frankfurt, Germany

**Keywords:** Acute heart failure, Cardiogenic shock, Extracorporeal circulation, ECLS, ECMO

## Abstract

**Objective:**

Extracorporeal life support (ECLS) is a life-saving procedure used in the treatment of severe cardiogenic shock. Within this retrospective single centre study, we examined our experience in this critically ill patient cohort to assess outcomes and clinical parameters by comparison of ECLS with or without selective left ventricular decompression.

**Methods:**

Between 2004 and 2014 we evaluated 48 adult patients with INTERMACS level 1 heart failure (age 49.7 ± 19.5 years), who received either central ECLS with (*n* = 20, 41.7%) or ECLS without (*n* = 28, 58.3%, including 10 peripheral ECLS) integrated left ventricular vent in our retrospective single centre trial.

**Results:**

Follow up was 100% with a mean of 0.83 ± 1.85 years. Bridge to ventricular assist device was feasible in 29.2% (*n* = 14), bridge to transplant in 10.4% (*n* = 5) and bridge to recovery in 8.3% (*n* = 4). Overall 30-day survival was 37.5%, 6-month survival 27.1% and 1-year survival 25.0%. ECLS support with left ventricular decompression showed favourable 30-day survival compared to ECLS without left ventricular decompression (*p* = 0.034). Thirty-day as well as long-term survival did not differ between the subgroups (central ECLS with vent, ECLS without vent and peripheral ECLS without vent). Multivariate logistic regression adjusted for age and gender revealed ECLS without vent as independent factor influencing 30-day survival.

**Conclusion:**

ECLS is an established therapy for patients in severe cardiogenic shock. Independent of the ECLS approach, 30-day mortality is still high but with superior 30-day survival for patients with ECLS and left ventricular venting. Moreover, by unloading the ventricle, left ventricular decompression may provide an important time window for recovery or further treatment, such as bridge to bridge or bridge to transplant.

## Introduction

Cardiogenic shock following acute or chronic heart failure is still associated with poor overall survival (Awad et al., 2012; Combes et al., 2008; Hochman et al., 1995; TRIUMPH Investigators et al., 2007). For critically ill patients with INTERMACS (Interagency Registry for Mechanically Assisted Circulatory Support) level 1 (“critical cardiogenic shock”) the use of veno-arterial extracorporeal life support (ECLS) is often the last therapeutic option for maintaining circulation and providing a longer window of time for further treatment. ECLS has proved its therapeutic value both during on-going cardio-pulmonary resuscitation and in the period immediately after successful cardio-pulmonary resuscitation, when end-organ function is severely impaired.

Patients with severe cardiogenic shock often present with both left and right heart failure. Low-output LV failure causes concomitant insufficient organ perfusion, while inadequate decompression of the failing left ventricle (LV) causes a backlog of blood into the lung with subsequent pulmonary congestion and a failure of the right ventricle (Weymann et al., 2014b). Especially in the setting of a dilated, poorly contracting heart with severe systolic dysfunction, decompression by LV venting might be crucial for the recovery of the heart muscle.

In the majority of ECLS cases, the right ventricle is decompressed sufficiently by the venous cannula, placed at the cavo-atrial boundary. If LV venting is not applied the LV is only decompressed if a patent foramen ovale or a septum defect with left to right shunt is present. Furthermore, it has been described that in the setting of severe systolic dysfunction (low cardiac output) with ECLS *in situ*, that ECLS itself can cause distension of the left ventricle, increasing preload and reducing subendothelial perfusion impairing myocardial ischemia (Einzig et al., 1980). These patho-mechanisms can be avoided by unloading of the LV using a vent (Aiyagari et al., 2006; Weymann et al., 2014b). It has been demonstrated that LV-decompression by venting improves lung, heart and end organ recovery with the ability to wean successfully from ECLS or to bridge to ventricular assist device or even to heart transplantation (Sandrio et al., 2014).

Several case reports and small case series have described the potential for LV decompression to improve outcome of patients in cardiogenic shock (Aiyagari et al., 2006; Hacking et al., 2015; Koenig et al., 1993; Ward et al., 1995). Kotani et al. (2013) investigated one of the biggest cohorts of children treated by veno-arterial ECLS with left-heart decompression (LA venting) to improve LV function (23 of 178 patients). Kotani reports the importance of early initiating of decompression for the probability for successful weanig from ECLS. However, these reports only describe the management in paediatric patients or in very small cohorts and valid data about the outcome of adult patients with or without LV decompression has not been published yet.

ECLS can be implanted either peripherally, usually by cannulation of the femoral, subclavian or axillary vessels, or centrally in combination with sternotomy. While peripheral ECLS (pECLS) by transcutaneously introducing venous and arterial cannulas into the target vessels is a simple and very fast technique, vessel injury, major bleeding and limb ischemia are common complications (Bisdas et al., 2011; Zimpfer et al., 2006). Today bypass techniques are established to reduce limb ischemia distal of the arterial cannulation. Peripheral ECLS with femoral vessel cannulation generates non-physiological retrograde arterial flow with the consequence that oxygenated blood may not reach coronary system or supra-aortic vessels (Secker-Walker et al., 1976). By comparison, cannulation of the axillary and subclavian artery is more time-consuming, requires (usually) a surgeon and vessels must be dissected carefully prior cannulation. In contrast to a fast peripheral approach, central ECLS (cECLS) therapy is more invasive and requires a surgical setting with thoracotomy. But cECLS generates a physiological antegrade flow support without the risk of peripheral vessel complications or under-perfusing organs. Although both techniques are highly standardized and logistics as well as equipment are available widely across specialized advanced heart failure units (Tschierschke, Katus & Raake, 2013), insufficient recovery of the ventricles post-ECLS implantation remains a crucial complication of this therapy (Aiyagari et al., 2006).

The aim of this single centre study was to analyse the outcome of patients receiving peripheral and central ECLS and to evaluate whether decompression of the failing left ventricle by vent implantation has a positive influence on outcomes, with the view to optimize the therapeutic strategy in this complex patient cohort.

## Materials and Methods

### Patients

#### Study population

Single-centre retrospective data was analysed of all patients with INTERMACS level 1 (cardiogenic shock with or without respiratory failure) who underwent ECLS implantation in our department between April 2004 and February 2014. The study design was approved by our institutional review board (Medical Faculty of the University Heidelberg, No. S-099/2015). Collectively, 48 adult patients with complete records could be identified (mean age 49.7  ± 19.5 years). Most common aetiology of cardiogenic shock was dilated cardiomyopathy (*n* = 10, 22.8%), followed by acute myocarditis (*n* = 9, 18.8%) and acute myocardial infarction (*n* = 6, 12.5%, Table 1). The majority received cECLS (with left ventricular decompression *n* = 20, 41.7%; without *n* = 18, 37.5%) whereas only 20.8% (*n* = 10) obtained pECLS. All pECLSs were implanted by cardiologists on the Cardiology Intensive Care unit (CCU) and none of the pECLS patients received a LV vent catheter. If patients were not in clinical stable conditions and therefore ground transportation not reasonable (departments are located in separate buildings without immediate connection) patients received pECLS on CCU. All patients treated at the Department of Cardiac Surgery received a cECLS. In addition, it became standard practice to add in left ventricular decompression in recent years. However, at the beginning of our study period individual surgeon preference determined whether a left ventricular decompression was applied or not. In our cohort, all LV vent catheters were implanted at time of initiation of ECLS.

**Table 1:** Aetiology of underlying disease.

Aetiology (*n* = 48)	
DCMP	20.8% (*n* = 10)
Acute myocarditis	18.8% (*n* = 9)
Myocardial infarction	12.5% (*n* = 6)
Aortic heart disease	10.4% (*n* = 5)
ICMP	6.3% (*n* = 3)
Others	31.3% (*n* = 15)

**Notes.**

Data are presented as percentage (*n*). DCMP, dilated cardiomyopathy.

ICMP ischemic cardiomyopathy

The following clinical indicators for cardiogenic shock were applied (“Fast-Entry-Criteria”): low cardiac index (CI) (<2.2 litres/min/m2), low systolic pressure (<90 mmHg for longer than 30 min) and specific clinical indications of central and peripheral hypoperfusion (cold extremities, oliguria or altered mental condition), which are refractory to fluid substitution and intravenous inotropic support. We defined respiratory failure as acute hypoxemia, which is refractory to protective pulmonary ventilation (PaO2 < 8.0 kPa, PaCO2 > 6.7 kPa, pH < 7.2 at Fi02 1.00).

#### Surgical procedures

**Figure 1:**
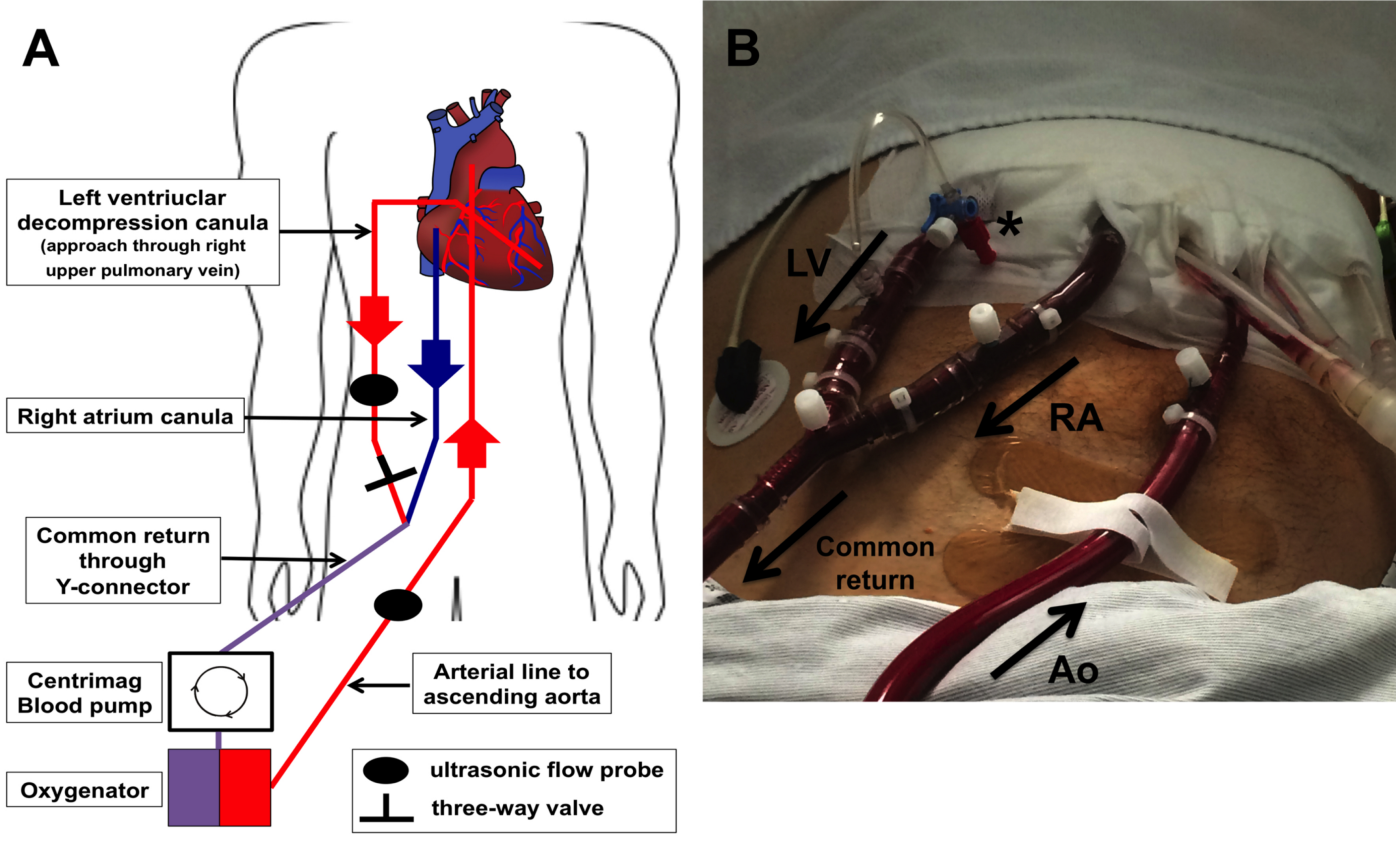
Illustration of central ECLS setup with LV vent (left ventricular). (A) Schematic illustration of cECLS (central extracorporeal life support) plus left ventricular decompression setup. (B) Postoperative picture showing ECLS assemblage on intensive care unit. LV decompression cannula (LV) is connected extracorporeal with the right atrial cannula (RA) to the common “venous” return of the circuit. A three-way valve is maintaining the ability to take blood gas samples of the LV blood to estimate lung perfusion as well as function. The aortic cannula (Ao) is implemented in the ascending aorta conducting the oxygenated blood back to the patient. Ultrasonic flow probes are applied on the outflow lines allowing flow metering.

Implantation of cECLS was performed as previously described (Weymann et al., 2014b). Central ECLS implantation was established using a median sternotomy approach for the cannulation of the ascending aorta. A 22 French arterial cannula was inserted using the Seldinger technique (Edwards Lifesciences Corporation, Irvine, CA, USA). Venous return was established by cannulation of the right atrium using a 28 French cannula (Medtronic, Minneapolis, MN, USA) and by directing the tip of the cannula towards the tricuspid valve. In case of left ventricular decompression, a heparin-coated 24 French cannula was inserted via the right superior pulmonary vein into the left ventricle. The right atrial and the left ventricular cannula were connected together (Y-connector) to the inflow of the ECLS system. Furthermore, the ECLS consisted of a Thoratec^®^ CentriMag blood pump (Thoratec^®^ CentriMag^®^ Blood Pump; Pleasanton, CA, USA) in combination with a D902 ECMO oxygenator (Dideco, Sorin Group, Milan, Italy). A detailed schematic illustration and postoperative picture of our cECLS setup is shown in Fig. 1.

We targeted an ECLS flow of 2.6 L/min/m^2^ body surface area. During the implantation procedure, transesophageal echocardiography (TEE) was performed routinely to obtain a correct position of all cannulas. Postoperatively, echocardiography was re-performed daily during intensive care unit (ICU) stay to ensure sufficient decompression of the left ventricle and to evaluate the ability of weaning. The LV decompression was always applied at the time of ECLS initiation.

In case of peripheral cannulation, cannulas were inserted transcutaneousely into the femoral artery (16 or 18 French OptiSite Arterial perfusion cannula, Edwards Life Science, Irvine CA, USA) and femoral vein (RAP femoral venous cannula 22 or 23/25 French; Sorin Group, Arvada, CO, USA) using the Seldinger technique. The position of the venous cannula (multi-drain cannula) was controlled by transesophageal echocardiography. Support was given by a portable centrifugal pump heart-lung support (Cardio Help; Maquet, Rastatt, Germany and Bio-Medicus^®^; Medtronic, Meerbusch, Germany). Continuous monitoring of ECLS flow was secured by using ultrasonic probes (Transonics Inc.). Ideal oxygenation and decarboxylation was measured in short intervals using point-of-care blood gas measurement. In addition, continuous brain oximetry (near-infrared spectroscopy, INVOS; Covidien, Mansfield MA, USA) was monitored on ICU. By using the three-way-tap in the proximal venous line and the LV-decompression line, selective blood gas analyses from the left heart can detect left ventricular and pulmonary recovery. Increased blood flow of the left ventricular cannula with greater oxygen saturation enabled additional documentation of lung recovery.

#### Weaning procedure

Feasibility of weaning from ECLS was determined by evaluating the following: (i) possibility of intermittent reduction of the ECLS blood flow to a minimum of 1/3 individual cardiac output (ii) preserved lung function (Horovitz Index > 200 mm Hg) with inspiration pressure at ventilator <30 cm H_2_O (iii) no signs of right ventricular failure (central venous pressure < 17 cm H_2_O) (iv) pulsatile systolic arterial blood pressure of >90 mm Hg under only low dosage of inotropic support for at least twelve hours (v) serum lactate levels <3 mmol/l and (vi) central venous oxygen saturation >65%. If LV vent was implanted, left atrial blood flow and gas analysis were evaluated.

#### Statistics

The study cohort was divided into two study groups: patients who underwent ECLS with LV venting and ECLS without LV venting. Furthermore, cECLS patient were divided into 2 subgroups, cECLS with and cECLS without LV venting for sub-analysis. Continuous variables are presented as mean ± standard deviation or as median combined with range. Categorical data are shown as percentage. Differences in preoperative, operative and postoperative data were elaborated by Student’s *t* test and chi-square test. Survival function was calculated by use of Kaplan–Meier estimator and differences in survival were determined using log rank test. Multivariate logistic regression model (stepwise and backward likelihood ratio selection) was performed to determine the influence of vent support on 30-day survival. To adjust the estimated benefit by venting we selected a priori the covariates age and gender. Two-tailed probability values less than 0.05 were considered as significant and values less than 0.01 as highly significant. Statistical analysis was performed using common statistic software (IBM SPSS 22.0, Chicago, IL, USA).

## Results

### Baseline characterization and duration of support

Follow up was completed successfully in 100%, with a mean follow up of 269 days (standard deviation 629 days, maximum 2,828 days). We distinguished between vent vs. no vent group as well as between pECLS and cECLS with and without vent (sub-analysis, see supplemental content). We saw a homogeneous distribution of sex between the study groups. Patients with ECLS and LV-vent were significantly younger and had lower body mass index than patients without LV-vent (38.28 vs. 57.9 years; *p* = 0.001 and 23.1 vs. 27.2 respectively, Table 2). Mean support on ECLS Patients was 6.10 ± 3.81 days. Length of ECLS support (*p* = 0.055), ventilation time (*p* = 0.091) and duration of in-hospital stay (*p* = 0.089) did not differ significantly between the study groups (ECLS with vent vs. ECLS without vent). Patients of the non-venting group more often had intra-aortic balloon pump support before ECLS was implemented (*p* = 0002, Table 2). Furthermore, the non-venting group contained more post-cardiotomy cases (*p* = 0.007).

**Table 2:** Baseline characteristic.

Baseline variables (*n* = 48)	w/ vent (*n* = 20)	w/o vent (28)	*p*-value
Female	6 (30)	11 (39)	0.507
Male	14 (70)	9 (50)	0.208
Age, years	38.3 ± 20.6	57.9 ± 14.0	<0.01
BMI kg/m2	23.1 ± 4.6	27.2 ± 4.5	0.004
Inotropic support preoperative	20 (100)	18 (100)	1.000
IABP pre ECLS	2 (10)	15 (54)	<0.01
Portable heart lung support system	7 (35)	2 (11)	0.015
Mechanical ventilation, pre op	14 (70)	9 (50)	0.208
ECLS post cardiotomy	2 (10)	13 (46)	<0.01

**Notes.**

Data are presented as *n* (percentage) or mean ± standard deviation (SD). Student *t* test for continuous variables or Chi-squared test for categorical variables. A probability value (*p*-value) of <0.05 was considered significant.

BMI Body Mass Index ECLS extracorporeal life support IABP intra-aortic balloon pump w/ with w/o without.

Central ECLS plus LV vent was in use for significantly longer than cECLS without LV venting (7.35 ± 4.16 vs. 4,78 ± 2.58 days; *p* = 0.030, see Table S2, supplemental content). Peripheral ECLS support was run for 6.0 ± 4.47 days, while there was no difference in support duration between pECLS and cECLS (6,13 ± 3.70 days; *p* = 0.924).

### Development of clinical parameters in the course of ECLS support

We evaluated liver, renal and lung function during extracorporeal life support. Specific points of time were: immediately prior to ECLS support, day one and day three on support or straight after ECLS explantation if weaning or bridging was possible. In our detailed analysis, we could not find relevant differences in end-organ function parameters between the groups vent vs. no vent. Sub-analysis revealed, that pre-implantation assessed Horovitz index was higher in the cECLS vent group compared to no vent group (214.21 ± 128.39 vs. 109.38 ± 63.04, *p* = 0.019, see Table S4, supplemental content), whereas at the same time the number of patients on respirator support was higher within this group. However, by definition, the majority of the patients suffered from a respiratory distress syndrome with a Horovitz index less than 200 mmHg (ARDS Definition Task Force et al., 2012).

### ECLS as therapeutic bridge

Four patients (8.3%) were successfully bridged to recovery by cECLS (one with vent and 3 without vent). The majority of survivors were bridged to VAD Systems (ventricular assist device, *n* = 14, 29.2%), whereas 6 (12.5%) patients underwent heart transplantation over the course of time (5 patients were bridged to transplant and 1 patients was bridged to VAD and then bridged to transplant, Table 3). Patients with LV-venting were more frequently bridged to VAD (bridge to bridge, *p* < 0.01, Table 3). Moreover, mortality rate under ECLS support was higher in patients without LV venting (*p* = 0.027). Sub-analysis revealed, that patients with cECLS and additional vent implantation were more frequently bridged to a VAD system than patients with cECLS without venting (50.0% vs. 5.6%, *p* ≤ 0.01).

**Table 3:** Outcome variables.

Outcome variables (*n* = 48)	w/ vent (*n* = 20)	w/o vent (28)	*p*-value
Length of support, days	7.4 ± 4.2	5.2 ± 3.4	0.055
Hospital stay, days	54.2 ± 62.71	28.9 ± 34.9	0.089
Mechanical ventilation, hours	761.3 ± 1,047.8	383.5 ± 426.4	0.091
Bridge to recovery	1 (5)	3 (11)	0.442
Bridge to transplant	4 (20)	1 (4)	0.146
Bridge to bridge	10 (50)	4 (14)	<0.01
Exitus during support	5 (25)	16 (57)	0.027
30-day survival	11 (55)	7 (25)	0.034
6-month survival	8 (40)	5 (18)	0.110
1-year survival	7 (35)	5 (18)	0.198
ECLS post cardiotomy	2 (10)	13 (46)	<0.01

**Notes.**

Data are presented as *n* (percentage) Chi-squared test for categorical variables. A probability value (*p*-value) of <0.05 was considered significant.

ECLS extracorporeal life support w/ with w/o without

### Left ventricular venting improves short-term survival

**Figure 2:**
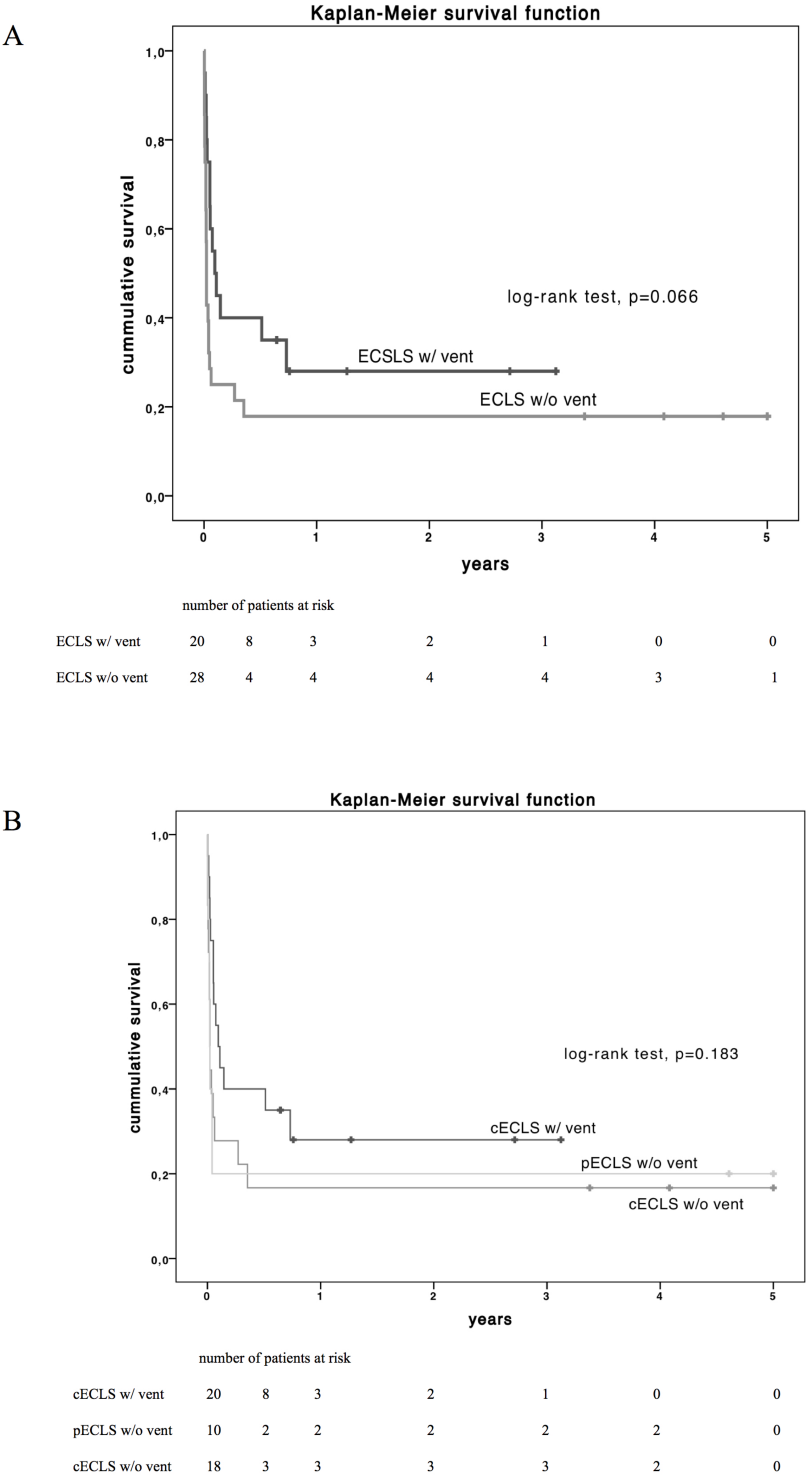
Estimated Kaplan–Meyer (KM) survival function (log-rank test) of the total cohort diversified by vent implantation (A) and by type of ECLS support (B) over 5-year follow up. cECLS w/ vent vs. cECLS w/o vent, *p* = 0.102; pECLS w/o vent vs. cECLS w/ vent, *p* = 0.117 and pECLS w/o vent vs. cECLS w/o vent, *p* = 0.950 and peripheral ECLS vs. central ECLS, *p* = 0.345. cECLS, central extracorporeal life support; pECLS, peripheral extracorporeal life support; w/, with; w/o, without.

Beyond organ function and clinical parameters, the principal purpose of ECLS in cardiogenic shock is the improvement of survival. Overall thirty-day survival was 37.5% (*n* = 18) in the overall study population. Patients with LV decompression by venting had a superior short-term survival after 30-days (55% vs. 25%, *p* = 0.034,) but no favourable survival after 6 and 12 months (*p* = 0.110 and 0.198 respectively, Table 3). Concerning long-term survival by Kaplan–Meier estimation, patients with LV vent showed a trend but no significant superiority (log-rank test *p* = 0.066, Fig. 2A) compared to patients without LV decompression. In addition, long-term survival did not differ between subgroups pECLS, cECLS without and cECLS with vent (*p* = 0.183, Fig. 2B).

### No-vent support as independent risk factors for 30-day mortality

Multivariate logistic regression model adjusted for age and gender revealed ECLS without vent as an independent risk factor for 30-day mortality (Odds ratio 3.667, 95% CI [1.074–12.518], *p* = 0.038, Table 4).

**Table 4:** Multivariate logistic regression model for 30-day mortality.

Variable	*p*-value	OR (95% CI)
ECLS w/o vent	0.038	3.667 (1.074–12.518)
Age	0.089	1.028 (0.996–1.016)
Male gender	0.815	0.864 (0.253–2.953)

**Notes.**

Multivariate logistic regression model for analysis of 30-day mortality adjusted for age and gender. Stepwise and backward likelihood ratio selection. A probability value (*p*-value) of <0.05 was considered as significant.

CI confidence interval ECLS w/o vent extracorporeal life support without venting OR Odds ratio

## Discussion

Independent of the underlying disease, acute therapy of cardiogenic shock remains a challenge for all disciplines involved. Taking into account, that conservatively treated cardiogenic shock is associated with a tremendously high mortality, ECLS therapy, if available, remains often the last chance for recovery (Mohite et al., 2014). ECLS utilization has increased in recent years, monitored by the ECLS Registry group, showing an acceptable overall survival of 44% (Paden et al., 2013). Our data represent nearly identical over all with a 30-day survival of 37.5%. Altogether, survival of patients in cardiogenic shock has improved significantly over the last decades, mainly due to advances in medical treatment, revascularization techniques, intensive care and mechanical support. Goldberg et al. (2009) reported improving trends in the hospital prognosis of patients with acute myocardial infarction and associated cardiogenic shock in a large three-decade-long retrospective study (from 1975 to 2005) with 13,663 patients.

In general, ECLS therapy without LV decompression is mainly limited by lung failure (pulmonary oedema with impaired oxygenation) and unrecognized left ventricular distension resulting in ventricular pressure overload, restricted coronary blood flow and insufficient cardiac recovery. The importance of left side decompression in ECLS has been described before. However, these publications report the use of balloons, blades or stentings to achieve an interatrial left to right side shunt (Haynes et al., 2009; Seib et al., 1999). Aiyagari et al. (2006) published their promising experience within a small cohort treated with a selective trans-septal left atrial cannulation to drain blood into the ECLS circuit. When evaluating mortality rates, death after cardiogenic shock occurs early even if ECLS support is given. In our cohort, 62.5% (*n* = 30 out of 41 decedents after one year) did not survive 30 days after ECLS implantation. Patients suffering from cardiogenic shock are by definition critically unwell and haemodynamically decompensated, with subsequently lung and end-organ dysfunction leading to fatal multi-organ failure.

End-organ dysfunction is the result of three obvious risk factors. The first is hypoxemia, itself a result of lung failure due to an impediment of the failing LV (or isolated/combined right heart failure), causing destruction and apoptosis of parenchymal cells. Secondly ischemia, due to reduced organ perfusion and finally congestion due to increased venous backflow.

Clinical parameters such as renal and liver function as well as parameters describing the lung function all failed to explain the beneficial outcome.

An important advantage of ECLS is the ability to instantly and continuously measure the blood flow crossing the lungs by Doppler-ultrasound measurement and pO2 of the LV decompressing cannula is of vital importance in terms of evaluation and timing of a potential weaning from ECLS or bridging to a further long-term support (e.g., VAD or transplant). These sophisticated options for heart and lung function evaluation allowed a more accurate timing to wean and are an important key to keep ECLS support duration as short as possible, but as long as required.

The optimal approach for utilizing ECLS remains debatable. The central approach via sternotomy allows a fast and excellent access to both right and left atrium as well as the ascending aorta. However, the surgical trauma is immense and early postoperative mobilization is limited. By comparison, peripheral support is of growing interest, due to a simple approach and a pervasively available technique in intensive care units without the need of immediate surgical support (Basra, Loyalka & Kar, 2011; Kar et al., 2012; Kar et al., 2011). At present, peripheral support is associated with complications like limb ischemia (Bisdas et al., 2011; Foley et al., 2010) and insufficient supply of oxygenated blood to both, the heart and the brain (Bisdas et al., 2011; Secker-Walker et al., 1976; Werdan et al., 2014). Moreover, retrograde flow is associated with a greater risk of aortic root thrombosis possibly resulting in cerebral insult and/or peripheral embolism, especially if no ejection is provided by the LV itself (Leontiadis et al., 2010) and an impediment of the weaning procedure due to the counter flow if support is reduced. However, if peripheral support is favoured or central support is not possible because of missing resources or logistics, we suggest setting up a transcutaneous left side decompression as well. Therefore, LV decompression could be accomplished by placing a transvenous cannula crossing the interatrial septum into the left atrium and connected to the venous return as previously described. Other groups have described the use of a trans-aortic decompression to unload the left ventricle in addition to a veno-arterial ECLS (Beurtheret et al., 2012; Koeckert et al., 2011). We are not in favour of additional trans-aortic decompression as first line procedure, mainly due to one very important limitation of this setting (if pECLS is implanted through the femoral vessels). The antegrade flow of the trans-aortic decompression catheter (usually implanted through the femoral artery) and the retrograde flow of the pECLS system are opposing and create non-predictable organ perfusion. In worst case this setting might even create a state of no-flow with need for increased vasopressor support. Moreover, while not important clinically, the costs of using two devices is significantly higher.

### LV vent should be applied to improve ECLS outcome

Our analysis demonstrated that ECLS without venting has a significantly inferior outcome after 30 days compared to ECLS with venting. Furthermore, mortality rate was significantly lower in patients with LV venting during ECLS support and non-ventilatory support was determined as strong independent risk factor for 30-day mortality. Even though our sub-analysis of patients with cECLS did not reveal a definite survival benefit for LV venting, we propose a combination of cECLS approach with LV decompression to justify the increase of invasiveness compared to the simpler pECLS approach. Moreover, a de-novo sternotomy may be avoided by using alternative approaches. These techniques have been described before by Weymann et al. (2014a) reporting a minimal invasive technique and avoiding sternotomy by approaching the heart via left lateral thoracotomy. Using the lateral entry, an additional LV decompression cannula can be added easily and tunnelled as described. Combination of both procedures provides the advantages of central support, LV decompression and the benefits of avoiding sternotomy, allowing early extubation and mobilization. In order to reduce surgical trauma and to make surgical procedure more feasible the LVAD outflow graft can be anastomosed to cannulate the aortic line, enabling an easy switch to long-term support (Weymann, Simon & Popov, 2014c).

Within our department, the vast majority of central ECLS procedures were performed using a LV vent within recent years, as a consequence of our growing experience with ECLS. Furthermore, the favourable results of LV-venting may be biased or confounded by generally improved perioperative management. This includes the continuous and systematic development of surgical strategies as well as the more sophisticated management on intensive care unit prior and post ECLS implantation.

### Left ventricular venting in pECLS?

As presented in our data cECLS alone did not lead to improved outcomes compared to pECLS. In our opinion, LV-venting must be performed to justify the much more invasive cECLS procedure. However, at time of writing there are no large studies available presenting pECLS with LV decompression and comparing different strategies. If a pECLS is implemented, LV-decompression could also be achieved by draining left atrial volume via a trans-septal cannula by a transaortic Impella^®^ system. A randomized multi-center study is required to address these essential major questions on this topic: (i) Is a routine peripheral support a non-inferior option to maintain ECLS compared to the central approach? (ii) Which technique (and/or device application) of LV decompression is best in pECLS, in a scenario where the central approach is not favoured or applicable?

## Conclusion

ECLS is a powerful and oftentimes end-stage tool to overcome life threatening cardiogenic shock, regardless of the underlying disease. We were able to demonstrate the benefit of LV venting in terms of 30-day survival, the most critical time frame in the state of refractory cardiogenic shock, independently of underlying cause. If ECLS is implemented, we strongly recommend this be done alongside decompression of the left ventricle. The technique of a separate LV-decompression in cECLS allows sophisticated options to follow both, cardiac and pulmonary recovery. Cardiogenic shock remains a highly challenging condition to manage and morbidity and mortality outcomes remain poor. To improve survival and to allow for further treatment options, using all available resources including therapy-enhancing techniques like LV-decompression are a vital next step that require further data to support and qualify their use and optimisation.

### Limitations

Our study demonstrates a relatively high number of ECLS patients with a specific LV decompression technique and a complete follow up. However, this study is limited by the fact that we performed a retrospective single-center analysis containing patients with a large variability of underlying diseases causing cardiogenic shock. Furthermore, all pECLS were implanted by Cardiologist on the Cardiology Care Unit and no LV venting was applied in pECLS patients, which leads to a certain bias.

##  Supplemental Information

10.7717/peerj.3813/supp-1 Supplemental Information 1 Supplemental content

10.7717/peerj.3813/supp-2 Supplemental Information 2 Raw data

## Additional Information and Declarations

### Data Availability

The following information was supplied regarding data availability: The raw data has been uploaded as a Supplemental File.
