# Supplementary material for: Extracorporeal life support with left ventricular decompression—improved survival in severe cardiogenic shock: results from a retrospective study
Source: PeerJ. 2017 Sep 29;5:e3813. doi: 10.7717/peerj.3813 (PMC5624302; doi:10.7717/peerj.3813)
Supplement: Supplemental Information 1 [file peerj-05-3813-s001.docx]

**Supplemental content**

Table 1. Baseline variables of subgroups.

| Baseline variables (n=48) |  | **cECLS w/ (n=20)** | **cECLS w/o (n=18)** | **p - value** |  | **pECLS (n=10)** | **cECLS (n=38)** | **p - value** |
| --- | --- | --- | --- | --- | --- | --- | --- | --- |
| female |  | 6 (30) | 9 (50) | 0.208 |  | 2 (20) | 15  (39.5) | 0.252 |
| male |  | 14 (70) | 9 (50) | 0.208 |  | 8 (80) | 23 (60.5) | 0.208 |
| age, years |  | 38.28 ± 20.58 | 60.36 ± 13.75 | <0.01 |  | 53.40 ± 13.98 | 48.74 ± 20.72 | 0.411 |
| BMI kg/m2 |  | 23.13 ± 4.57 | 26.46 ± 4.21 | 0.028 |  | 28.50 ± 4.87 | 24.66 ± 4.66 | 0.027 |
| inotropic support preoperative |  | 20 (100) | 18 (100) | 1.000 |  | 10 (100) | 38 (100) | 1.000 |
| IABP |  | 2 (10) | 8 (44) | 0.016 |  | 7 (70) | 10 (26.3) | 0.01 |
| portable heart lung support system |  | 7 (35) | 2 (11) | 0.084 |  | 0 (0) | 9 (23.7) | 0.088 |
| mechanical ventilation, pre op |  | 14 (70) | 9 (50) | 0.208 |  | 10 (100) | 23 (60.5) | 0.017 |
| ECLS post cardiotomy |  | 2 (10) | 11 (61.1) | <0.01 |  | 2 (20) | 13  (34.2) | 0.388 |
| Data are presented as n (percentage) or mean ± standard deviation (SD). Student t test for continuous variables or Chi-squared test for categorical variables. A probability value (p-value) of <0.05 was considered significant. BMI, Body Mass Index; C, central; ECLS, extracorporeal life support; IABP, intra-aortic balloon pump; p, peripheral, w/ , with; w/o, without. | | | | | | | | |

Table 2. Outcome variables of subgroups.

| **Outcome variables (n=48)** |  | **cECLS w/ vent (n=20)** | **cECLS w/o vent (n=18)** | **p - value** |  | **pECLS (n=10)** | **cECLS (n=38)** | **p - value** |
| --- | --- | --- | --- | --- | --- | --- | --- | --- |
| length of support, days |  | 7.4 ± 4.2 | 4.8 ± 2.6 | 0.030 |  | 6.0 ± 4.5 | 6.1 ± 3.7 | 0.924 |
| hospital stay, days |  | 54.3 ± 62.7 | 29.2 ± 37.00 | 0.157 |  | 28.5 ± 32.9 | 41.3 ± 51.9 | 0.466 |
| mechanical ventilation, hours |  | 761.3 ± 1047.8 | 372.6 ± 406.2 | 0.149 |  | 403.1 ± 482.7 | 577.13 ± 823.6 | 0.399 |
| bridge to recovery |  | 1 (5) | 3 (16.7) | 0.242 |  | 0 (0) | 4 (10.5) | 0.284 |
| bridge to transplant |  | 4 (20) | 0 (0) | 0.107 |  | 1 (10) | 4 (1) | >0.999 |
| bridge to bridge |  | 10 (50) | 1 (5.6) | <0.01 |  | 3 (30) | 11 (28.9) | 0.948 |
| exitus during support |  | 5 (25) | 10 (56) | 0.054 |  | 6 (60) | 15 (39.5) | 0.244 |
| 30-day survival |  | 11 (55) | 5 (27.8) | 0.090 |  | 2 (20) | 16 (42.1) | 0.199 |
| 6-month survival |  | 8 (40) | 3 (16.7) | 0.160 |  | 2 (20) | 11 (28.9) | 0.710 |
| 1-year survival |  | 7 (35) | 3 (16.7) | 0.278 |  | 2 (20) | 10 (26.3) | >0.999 |
| Data are presented as n (percentage) Chi-squared test for categorical variables. A probability value (p-value) of <0.05 was considered significant. C, central; ECLS, extracorporeal life support; p, peripheral; w/ , with; w/o, without. | | | | | | | | |

Table 3. Progress of clinical parameters following ECLS.

| **Progress of clinical paramteres (n=48)** | **w/ vent (n=20)** | **w/o vent (28)** | **p-value** | |  |
| --- | --- | --- | --- | --- | --- |
| **prä ECLS** |  |  |  | |  |
| creatinine mg/dl | 1.82 ±1.03 | 2.0± 1.0 | | 0.597 |  |
| GOT U/L | 1428.8 ± 2207.6 | 800.1  ± 1490.0 | | 0.260 |  |
| GPT U/L | 894.7 ±1414.63 | 653.9 ± 1396.1 | | 0.573 |  |
| lactate mg/dl | 70.8 ± 47.1 | 75.1 ± 35.2 | | 0.853 |  |
| FiO2 % | 89.8 ± 15.2 | 90.4 ± 19.4 | | 0.919 |  |
| paO2 mmHg | 189.6 ± 110.8 | 150.5 ± 65.4 | | 0.158 |  |
| pCO2 mmHg | 37.3 ± 11.10 | 39.4 ± 6.8 | | 0.451 |  |
| Horovitz mmHg | 214.24 ± 128.4 | 146.4 ± 96.3 | | 0.126 |  |
| **after one day support** |  |  | |  |  |
| creatinine mg/dl | 1.71 ± 1.05 | 1.80 ± 0.73 | | 0.757 |  |
| bilirubine total mg/dl | 5.23 ± 4.63 | 5.0 ± 3.9 | | 0.870 |  |
| GOT U/L | 1522.8 ± 2516.6 | 2146.0 ± 4005.0 | | 0.558 |  |
| GPT U/L | 693.3 ± 1063.2 | 753.9 ± 1640.6 | | 0.890 |  |
| lactate mg/dl | 28.4 ± 24.1 | 43.3 ± 36.0 | | 0.853 |  |
| FiO2 % | 59.2± 19.0 | 62.9 ± 28.4 | | 0.634 |  |
| paO2 mmHg | 1805 ±65.3 | 178.1 ±73.8 | | 0.721 |  |
| paCO2 mmHg | 38.07 ± 4.0 | 39.4 ± 5.5 | | 0.373 |  |
| Horovitz Index mmHg | 352.6 ± 206.6 | 362.1 ± 201.0 | | 0.909 |  |
| **after three days support** |  |  |  | |  |
| creatinine mg/dl | 1.69 ± 1.26 | 1.53 ± 0.62 | 0.642 | |  |
| bilirubine total mg/dl | 5.5 ± 6.4 | 5.93 ± 6.83 | 0.846 | |  |
| GOT U/L | 559.4 ± 885.3 | 807.3 ± 1018.6 | 0.436 | |  |
| GPT U/L | 397.5 ± 554.5 | 408.07 ± 736.3 | 0.959 | |  |
| lactate mg/dl | 15.1 ± 14.0 | 19.3 ± 10.6 | 0.319 | |  |
| paO2 mmHg | 180.1 ± 49.8 | 54.5 ± 22.1 | 0.346 | |  |
| paCO2 mmHg | 38.5 ± 4.5 | 164.3 ±50.6 | 0.949 | |  |
| FiO2 % | 57.5 ± 19.0 | 38.6 ± 5.6 | 0.646 | |  |
| Horovitz Index mmHg | 376 ± 112.03 | 416.21 ± 261.2 | 0.608 | |  |
| **post ECLS** |  |  |  | |  |
| creatinine mg/dl | 1.69 ± 1.26 | 1.49 ± 0.52 | 0.275 | |  |
| bilirubine total mg/dl | 5.51 ± 6.42 | 4.9 ± 6.7 | 0.417 | |  |
| GOT U/L | 559.39 ± 885.26 | 390.8 ± 710.7 | 0.430 | |  |
| GPT U/L | 397.5 ± 554.54 | 312.5 ± 626.9 | 0.491 | |  |
| lactate mg/dl | 15.13 ± 14.03 | 20.9 ± 22.2 | 0.404 | |  |
| paO2 mmHg | 180.11 ± 49.84 | 137.3± 50.7 | 0.264 | |  |
| paCO2 mmHg | 38.46 ± 4.45 | 44.1 ± 13.7 | 0.436 | |  |
| FiO2 % | 57.5 ± 18.962 | 62.8 ± 13.9 | 0.134 | |  |
| Horovitz Index mmHg | 376 ± 112.03 | 260.5 ± 82.2 | 0.308 | |  |
| Data are presented as mean ± standard deviation (SD). Student t test for continuous variables. A probability value (p-value) of <0.05 was considered significant. ECLS, extracorporeal life support; FiO2, fraction of inspired oxygen; Horovitz index, PaO2 ([partial pressure of oxygen](https://galileo.kgu.de/owa/redir.aspx?SURL=P_QZGK7FPZWaqkzROgblXIddmuJh1CJLEDIjZwhUKA8fXNwzKhjUCGgAdAB0AHAAOgAvAC8AZQBuAC4AdwBpAGsAaQBwAGUAZABpAGEALgBvAHIAZwAvAHcAaQBrAGkALwBQAGEAcgB0AGkAYQBsAF8AcAByAGUAcwBzAHUAcgBlAF8AbwBmAF8AbwB4AHkAZwBlAG4A&URL=http%3a%2f%2fen.wikipedia.org%2fwiki%2fPartial_pressure_of_oxygen) in blood) / FiO2; GOT, glutamic oxaloacetic transaminase; GPT, glutamate-pyruvate transaminase; paO2, partial pressure of arterial oxygen; paCO2, partial pressure of arterial carbon dioxide; w/ , with; w/o, without. | | | | | |

Table 4. Progress of clinical parameters (subgroups) following ECLS.

| **progress of variables (n=48)** | **cECLS w/ vent**  **(n=20)** | **cECLS w/o vent (n=18)** | **p- value** | |  | **pECLS (n=10)** | **cECLS (n=38)** | **p-value** |
| --- | --- | --- | --- | --- | --- | --- | --- | --- |
| **prä ECLS** |  |  |  | |  |  |  |  |
| creatinine mg/dl | 1.82 ±1.03 | 1.861 ± 1.42 | | 0,916 |  | 2.25 ± 0.94 | 1.84 ± 1.21 | 0.321 |
| GOT U/L | 1428.8 ± 2207.6 | 506.1  ± 1072.7 | | 0,117 |  | 1354.7 ± 2026.6 | 993.3 ± 1802.9 | 0,602 |
| GPT U/L | 894.7 ±1414.6 | 356.4 ± 768.8 | | 0,161 |  | 1216.0 ± 2089.3 | 640.5 ± 1172.1 | 0,446 |
| lactate mg/dl | 70.8 ± 47.1 | 80.8 ± 36.4 | | 0,699 |  | 58.0 ± 35.4 | 76.3 ± 39.7 | 0,558 |
| FiO2 % | 89.8 ± 15.2 | 96.9 ± 10.1 | | 0,127 |  | 78.9 ± 26.6 | 93.2 ± 13.3 | 0,151 |
| paO2 mmHg | 189.6 ± 110.8 | 155.4 ±75.3 | | 0.311 |  | 141.8 ± 45.4 | 1723.0 ±95.4 | 0.176 |
| pCO2 mmHg | 37.3 ± 11.1 | 39.5 ± 6.5 | | 0.514 |  | 39.4 ±7.6 | 38.4 ±9.1 | 0.751 |
| Horovitz mmHg | 214.2 ± 128.4 | 109.4 ± 63.04 | | 0,019 |  | 195.7 ± 115.9 | 176.1 ± 119.1 | 0,723 |
| **after one day support** |  |  | |  |  |  |  |  |
| creatinine mg/dl | 1.71 ± 1.05 | 1.73 ± 0.87 | | 0,966 |  | 1.94 ± 0.33 | 1.72 ± 0.95 | 0,529 |
| bilirubine total mg/dl | 5.2 ± 4.6 | 5.1 ± 4.1 | | 0,910 |  | 4.9 ± 3.9 | 5.1 ± 4.3 | 0,891 |
| GOT U/L | 1522.8 ± 2516.6 | 2244.6 ± 4568.9 | | 0,558 |  | 1948.9 ± 2811.2 | 1852.8 ± 3563.1 | 0,944 |
| GPT U/L | 693.3 ± 1063.2 | 715.0 ± 1865.4 | | 0,966 |  | 831.7 ± 1173.4 | 703.2 ± 1460.7 | 0,818 |
| lactate mg/dl | 28. ±4 24.1 | 49.8 ± 41.8 | | 0,699 |  | 30.4 ±15.3 | 37.9 ± 34.4 | 0,545 |
| FiO2 % | 59.2 ± 19.0 | 49.8 ± 41.8 | | 0,590 |  | 60.0 ± 21.6 | 61.4 ± 25.2 | 0,887 |
| paO2 mmHg | 170.5 ±65.3 | 174.7 ± 69.1 | | 0.852 |  | 184.9 ± 87.0 | 172.3 ± 66.1 | 0.649 |
| paCO2 mmHg | 38.1 ± 4.0 | 39.8 ± 5.11 | | 0.250 |  | 38.5 ± 6.5 | 38.86 ± 4.6 | 0.860 |
| Horovitz Index mmHg | 352.6 ± 206.6 | 358.1 ± 214.5 | | 0,952 |  | 373.2 ± 179.3 | 355.9 ± 206.1 | 0,864 |
| **after three days support** |  |  |  | |  |  |  |  |
| creatinine mg/dl | 1.69 ± 1.26 | 1.38 ± 0.55 | 0,441 | |  | 1.79 ± 1 .68 | 1.56 ± 1.03 | 0,581 |
| bilirubine total mg/dl | 5.5 ± 6.4 | 4.7 ± 5.0 | 0,703 | |  | 8.1 ± 9.3 | 5.2 ± 5.8 | 0,290 |
| GOT U/L | 559.4 ± 885.3 | 735.3 ± 1132.0 | 0,637 | |  | 930.9 ± 857.3 | 629.7 ± 976.3 | 0,458 |
| GPT U/L | 397.5 ± 554.5 | 386.8 ± 851.4 | 0,967 | |  | 446.3 ± 543.1 | 393.23 ± 674.7 | 0,848 |
| lactate mg/dl | 15.1 ± 14.0 | 17.7 ± 11.6 | 0,608 | |  | 22.0 ± 8.7 | 16.1 ± 13.0 | 0,262 |
| paO2 mmHg | 180.1 ± 49.8 | 150.33± 49.0 | 0.118 | |  | 188.29 ± 47.1 | 168.2 ± 50.9 | 0.347 |
| paCO2 mmHg | 38.5 ± 4.5 | 37.2 ± 5.0 | 0.460 | |  | 41.0 ± 6.3 | 37.9 ± 4.6 | 0.152 |
| FiO2 % | 57.5 ± 19.0 | 57.6 ± 25.9 | 0,992 | |  | 49.3 ± 13.7 | 57.5 ± 21.6 | 0,342 |
| Horovitz Index mmHg | 376 ± 112.0 | 386.8 ± 301.1 | 0,921 | |  | 469.2 ± 186.9 | 380.6 ± 207.9 | 0,393 |
| **post ECLS** |  |  |  | |  |  |  |  |
| creatinine mg/dl | 1.02 ± 0.92 | 1.51 ± 0.56 | 0,294 | |  | 1.36 ± 0 | 1.33 ± 0.71 | 0.967 |
| bilirubine total mg/dl | 8.73 ± 9.2 | 5.80 ± 7.5 | 0,578 | |  | 2.0 ± 0.6 | 6.9 ± 7.8 | 0.409 |
| GOT U/L | 90.75 ± 40.36 | 374.00 ± 765.91 | 0,489 | |  | 508.00 ± 0 | 271.0 ± 610.6 | 0.718 |
| GPT U/L | 82.3 ± 58.6 | 314.4 ± 677.1 | 0,520 | |  | 299  ± 0 | 230.00 ± 538.4 | 0.905 |
| lactate mg/dl | 10.8 ± 5.8 | 13.51 ± 3.77 | 0,360 | |  | 46.1 ± 46.9 | 12.5 ± 4.5 | 0.495 |
| paO2 mmHg | 171.0 ±38.2 | 154.14 ± 39.6 | 0.510 | |  | 160.3 ± 38.1 | 78.5 ± 47.4 | 0.020 |
| paCO2 mmHg | 38.2 ± 6.3 | 40.5 ± 4.4 | 0.501 | |  | 57.0 ± 31.3 | 39.66 ± 5.0 | 0.576 |
| FiO2 % | 50.0 ± 10.8 | 61.4 ± 12.2 | 0,154 | |  | 67.50 ± 24.8 | 57.3 ± 12.5 | 0.663 |
| Horovitz Index mmHg | 328.0 ± 97.2 | 267.8 ± 89.7 | 0,406 | |  | 224.0 ± 0 | 290.4 ± 90.9 | 0.513 |
| Data are presented as mean ± standard deviation (SD). Student t test for continuous variables. A probability value (p-value) of <0.05 was considered significant. C, central; ECLS, extracorporeal life support; FiO2, fraction of inspired oxygen; Horovitz index, PaO2 ([partial pressure of oxygen](https://galileo.kgu.de/owa/redir.aspx?SURL=P_QZGK7FPZWaqkzROgblXIddmuJh1CJLEDIjZwhUKA8fXNwzKhjUCGgAdAB0AHAAOgAvAC8AZQBuAC4AdwBpAGsAaQBwAGUAZABpAGEALgBvAHIAZwAvAHcAaQBrAGkALwBQAGEAcgB0AGkAYQBsAF8AcAByAGUAcwBzAHUAcgBlAF8AbwBmAF8AbwB4AHkAZwBlAG4A&URL=http%3a%2f%2fen.wikipedia.org%2fwiki%2fPartial_pressure_of_oxygen) in blood) / FiO2; GOT, glutamic oxaloacetic transaminase; GPT, glutamate-pyruvate transaminase; paO2, partial pressure of arterial oxygen; paCO2, partial pressure of arterial carbon dioxide; p, peripheral; w/ , with; w/o, without. | | | | | | | | |
